# Transplantation of genetically engineered cardiac fibroblasts producing recombinant human erythropoietin to repair the infarcted myocardium

**DOI:** 10.1186/1755-1536-1-7

**Published:** 2008-11-03

**Authors:** Emil Ruvinov, Orna Sharabani-Yosef, Arnon Nagler, Tom Einbinder, Micha S Feinberg, Radka Holbova, Amos Douvdevani, Jonathan Leor

**Affiliations:** 1 Neufeld Cardiac Research Institute, Sheba Medical Center, Tel-Aviv University, Tel-Hashomer, Israel; 2 Institute of Hematology, Sheba Medical Center, Tel-Hashomer, Israel; 3 Department of Nephrology, Soroka University Medical Center, Ben-Gurion University, Beer-Sheva, Israel

## Abstract

**Background:**

Erythropoietin possesses cellular protection properties. The aim of the present study was to test the hypothesis that *in situ *expression of recombinant human erythropoietin (rhEPO) would improve tissue repair in rat after myocardial infarction (MI).

**Methods and results:**

RhEPO-producing cardiac fibroblasts were generated *ex vivo *by transduction with retroviral vector. The anti-apoptotic effect of rhEPO-producing fibroblasts was evaluated by co-culture with rat neonatal cardiomyocytes exposed to H_2_O_2_-induced oxidative stress. Annexin V/PI assay and DAPI staining showed that compared with control, rhEPO forced expression markedly attenuated apoptosis and improved survival of cultured cardiomyocytes. To test the effect of rhEPO on the infarcted myocardium, Sprague-Dawley rats were subjected to permanent coronary artery occlusion, and rhEPO-producing fibroblasts, non-transduced fibroblasts, or saline, were injected into the scar tissue seven days after infarction. One month later, immunostaining identified rhEPO expression in the implanted engineered cells but not in controls. Compared with non-transduced fibroblasts or saline injection, implanted rhEPO-producing fibroblasts promoted vascularization in the scar, and prevented cell apoptosis. By two-dimensional echocardiography and postmortem morphometry, transplanted EPO-engineered fibroblasts did not prevent left ventricular (LV) dysfunction and adverse LV remodeling 5 and 9 weeks after MI.

**Conclusion:**

*In situ *expression of rhEPO enhances vascularization and reduces cell apoptosis in the infarcted myocardium. However, local EPO therapy is insufficient for functional improvement after MI in rat.

## Background

Erythropoietin (EPO), a hematopoietic cytokine, has been shown to possess cardioprotective characteristics that can minimize ischemic injury and improve myocardial viability and function [1]. Systemic administration of recombinant human EPO (rhEPO) before or after myocardial ischemia reduces infarct size and improves cardiac function [2,3]. The beneficial effects of EPO are mediated by apoptosis inhibition, driven by Akt/phosphoinositide3-kinase signaling [4,5], and neovascularization by stimulation of endothelial progenitor cells [6,7]. However, systemic administration of EPO to patients with coronary artery disease could lead to adverse effects, such as high viscocity, impaired tissue perfusion, high blood pressure and an increased incidence of thrombosis [8,9]. Hematocrit elevation has been linked to excess mortality in patients with ischemic heart disease [10,11]. Over-expression of rhEPO in transgenic mice resulted in cardiac dysfunction and reduced life span [12,13]. Overall, the potential adverse effects associated with systemic EPO administration suggest that alternative approaches are needed.

A particularly beneficial approach would be a strategy that could provide local rhEPO delivery into the infarcted heart. A recent study has suggested that cardiac fibroblasts are critical effectors for erythropoietin-mediated pro-survival signaling and cardiac protection [14]. In the present study, we aimed to test the hypothesis that cardiac fibroblasts can be reprogrammed *ex vivo *to produce rhEPO, and that re-implantation of the engineered cells into the infarcted myocardium could promote tissue healing and repair.

## Materials and methods

The study was performed in accordance with the Animal Care and Use Committee guidelines of the Tel Aviv University, Tel Aviv, Israel, which conforms to the policies of the American Heart Association.

### Retroviral vector

Amphotrophic rhEPO (pBabe-EPO) retrovirus-containing packaging cell line PT67 was used for retroviral vector production [15]. Twenty-four hour filtered (0.45 μm) culture virus-containing supernants were stored at -80°C for future experiments. The presence and titer of rhEPO-retroviral vector were assayed by infecting BALB/3T3 (ATCC number CCL-163) mouse fibroblast cell line and a CFU (colony-forming unit) number determination.

### Isolation of cardiac cells

Cardiac fibroblasts and cardiomyocytes were isolated from 1–2 day old neonatal Sprague-Dawley rats (Harlan Lab, Jerusalem, Israel) as previously described [16]. We used a 30 minute pre-plating procedure to obtain cardiac fibroblasts and to reduce the number of non-myocyte cells in cardiomyocyte culture. The purity of obtained cardiac fibroblast culture was confirmed microscopically by characteristic cell morphology. Neonatal rat cardiomyocytes were used for oxidative stress studies and cardiac fibroblasts were used for transduction and transplantation studies.

### Cell transduction

BALB/3T3 (ATCC number CCL-163) mouse fibroblast cell line and isolated cardiac fibroblasts were used for transduction experiments. RhEPO retrovirus-containing supernants were first incubated at room temperature with a Superfect cationic transfection reagent (QIAGEN, Westburg Ltd., Be'er-Sheva, Israel) to a final concentration of 10 μg/ml for 15 minutes [17]. Finally, the viral stock was added to 70–80% confluent BALB/3T3 or cardiac fibroblast culture. After 24 h incubation at 37°C, puromycin selection was applied (2.5 μg/ml). Transduction efficiency was approximately 80%, as evaluated qualitatively by relative confluence determination (before and after puromycin selection). Twenty-four hour culture supernants were collected for EPO assessment. Supernants were stored at -20°C for future experiments.

### Assessment of EPO production and activity

#### RhEPO protein levels

The presence of rhEPO in transduced culture supernants was analyzed by a commercial ELISA kit (R&D Systems, Minneapolis, MN, USA). EPO concentration was normalized to 10^6 ^cells for 24 h.

#### RhEPO biologic activity (TF-1 cell proliferation assay)

EPO biological activity was analyzed using the EPO-sensitive human erythroleukemia cell line TF-1, in which proliferation and metabolic activity is EPO-dependent [18].

TF-1 cells were seeded in 96-well plates (1 × 10^5 ^cell per well in 100 μl of RPMI medium). A total of 100 μl of previously collected culture supernants, controls or standards (0.01 to 5 IU/ml rhEPO; Eprex, Beerse, Belgium) were added to each well. On day 5 of the experiment, TF-1 relative cell number was analyzed using colorimetric XTT assay kit (Biological Industries, Beth Haemek, Israel). The XTT assay is based on the ability of metabolically active (live) cells to reduce tetrazolium salt (XTT) to orange-colored compounds of formazan. EPO activity in supernants from rhEPO-transduced fibroblast culture was calculated from XTT rhEPO standard calibration curve. EPO relative biological activity was determined by EPO activity level/EPO protein level (measured by ELISA) ratio. This amount-normalized protein ratio was used for comparison of biological activity between expressed EPO (produced from transduced cells) and recombinant protein.

### Oxidative stress

After 6 days in culture, isolated rat neonatal cardiomyocytes were pretreated with supernants collected from rhEPO-transduced or non-transduced (for control) cardiac fibroblasts for 24 h, after which H_2_O_2 _was directly added to culture medium to a 150 μM final concentration for 60 minutes, and the degree of apoptosis was tested.

### Apoptosis assays

#### Annexin V/propidium iodide assay

Apoptosis assessment was performed using Annexin-V-FLUOS (fluorescein)/propidium iodide (PI) staining kit (Roche Diagnostics GmbH, Penzberg, Germany). The samples were prepared according to the manufacturer's instructions and were analyzed by flow cytometry. Annexin-V-positive and PI-negative cells were scored as apoptotic cells.

#### DAPI staining

Apoptotic morphological changes were evaluated by DAPI (4',6'-diamidino-2-phenylindole) staining. After oxidative stress induction, cells were gently washed with phosphate-buffered saline (PBS) and fixed with cold methanol for 10 minutes. The plates were washed twice with PBS and incubated for 5 minutes with 300 μM DAPI (catalogue number D-3571, Molecular Probes, Eugene, Oregon, USA) at 37°C. Finally, the cells were washed three times with PBS and examined by fluorescence microscopy. Apoptotic cells were identified by condensation and fragmentation of the nuclei, and were quantified by counting a total of 200 nuclei from each well and calculating the percentage of apoptotic nuclei.

### Rat model of acute myocardial infarction

Male Sprague-Dawley rats (approximately 250 g; Harlan Lab, Jerusalem, Israel) were subjected to acute myocardial infarction (AMI) induction by permanent coronary artery occlusion, as previously described [19].

### Cell transplantation

RhEPO-transduced and non-transduced fibroblast transplantations were performed 7 days after AMI. After 10 days in culture (7 days after transduction, 3 passages), cardiac fibroblasts were prepared for transplantation by trypsinization with 0.25% Trypsin-EDTA solution for 1 minute at 37°C. The rhEPO expression level in prepared cells was 7.0 ± 0.23 mIU/24 h/10^6 ^cells. Following centrifugation (1,500 rpm, 10 minutes at room temperature) the cells were re-suspended in saline. The final cell density for implantation was 1 × 10^6 ^cells/100 μl. Rats were anesthetized and the chest was opened under sterile conditions. The infarcted area was identified visually by the surface scar and wall motion akinesis. Rats were randomized and selected either for injection of 1 × 10^6 ^rhEPO-transduced cells, 1 × 10^6 ^non-transduced cells, or saline (n = 5 in each group). All injections were made into the center of the scar. After the injection, the air was expelled from the chest, and the surgical incision was sutured closed [19,20].

### Echocardiography

Transthoracic echocardiography was performed on all animals before transplantation (baseline echocardiogram) and 5 and 9 weeks later (4 and 8 weeks post-transplantation, respectively). Echocardiograms were performed with a commercially available echocardiography system (Sonos 7500; Philips) equipped with 12.5 MHz phased-array transducer (Hewlett-Packard, Andover, MA, USA) as previously described [21,22]. All measurements were averaged over three consecutive cardiac cycles and were performed by an experienced technician who was blinded to the treatment group.

### Hematocrit measurements

For evaluation of possible systemic effects of rhEPO produced from transplanted cells, hematocrit measurements were performed before or 1 and 4 weeks after transplantation.

### Immunohistochemical analysis

Four weeks after transplantation, hearts were arrested with 15% KCl, perfused with formaldehyde 4% (15 mmHg) for 20 minutes, sectioned into 3–4 transverse slices, and parallel to the atrioventricular ring. Each slice was fixed with 10% buffered formalin, embedded in paraffin, and sectioned into 5 μm slices. Serial sections were stained with the following agents: anti-α-smooth muscle actin monoclonal antibodies (Sigma-Aldrich, Rehovot, Israel) to localize pericytes and arterioles for neovascularization evaluation; anti-active caspase-3 polyclonal antibodies (Biocare Medical, Walnut Creek, CA, USA) for apoptosis detection; anti-rhEPO monoclonal antibodies (R&D Systems, Minneapolis, MN, USA) to localize rhEPO production and distribution; lectin (*Bandeirea *simplicifolia agglutinin BS-1; Sigma-Aldrich, Rehovot, Israel) to localize vascular endothelium; and terminal deoxynucleotidyl transferase dUTP nick end labeling (TUNEL) using Apoptag Peroxidase In situ Apoptosis Kit (S7101, Chemicon, Temecula, CA, USA) to assess apoptosis. The degree of neovascularization was evaluated by capillary blood vessel and arteriole counts in the infarct border zone (at least three different fields were counted). Apoptosis evaluation was made by active caspase-3 or TUNEL-positive cell counts in the infarct border zone (at least five different fields were counted). Co-staining was made with haematoxylin or methyl green for TUNEL.

### Morphometric analysis

After perfusion pressure fixation (15 mmHg), slides stained with Masson's trichrome (Dexmore, Israel) were used to assess left ventricular (LV) remodeling by morhpometric analysis, as previously described [23]. The following parameters were measured: LV maximal diameter (mm), average wall thickness (mm; averaged from three measurements of septum thickness), average scar thickness (mm; averaged from three measurements of scar thickness), relative scar thickness (average scar thickness/average wall thickness), LV muscle area (mm^2^; including the septum), LV cavity area (mm^2^), whole LV area (mm^2^), infarct expansion index ([LV cavity area/Whole LV area]/Relative scar thickness), epicardial scar length (mm), and endocardial scar length (mm).

### Statistical analysis

All values are given as means ± standard error of the mean (SEM) from at least three independent experiments. RhEPO activity results were compared with Student's *t*-test for unpaired data. Cardiomyocyte or general cell apoptosis results and blood vessel counts were compared by one-way ANOVA with Bonferroni post test.

In the echocardiography study, because each animal was used as its own control, changes between baseline and 4 weeks in the control and treated groups were assessed with paired *t*-tests. Comparisons of the changes from baseline to 5 and 9 weeks in the control and treatment groups were made with repeated-measure two-way ANOVA. The ANOVA model included control versus treatment and baseline versus 5 and 9 weeks as factors, as well as the interaction between the two factors. GraphPad Prism version 4.00 for Windows (GraphPad Software, San Diego, California, USA) was used for analysis.

Comparison of morphometry parameters and hematocrit measurements among treatment groups was analyzed by one-way ANOVA with Bonferroni post test. All tests were performed using GraphPad Prism Version 4.0. *P *< 0.05 was considered statistically significant.

## Results

### *Ex vivo *retroviral gene transfer and rhEPO expression

To test retroviral ability for stable expression of biologically active rhEPO, the initial transduction experiments were carried out using BALB/3T3 mouse fibroblast cell line. Table 1 describes the activity of EPO produced from transduced cells. ELISA was used to evaluate EPO concentrations, and its biological activity was determined by proliferation of the EPO-sensitive human erythroleukemia cell line TF-1. The activity induced by recombinant human EPO (Eprex) was used as a standard in this assay. Produced EPO protein was found to be 35 times more biologically active than recombinant protein (Eprex; Table 1, *p *= 0.0006). The rhEPO production level in transduced cultures was 11.5 ± 0.8 mIU/24 h/10^6 ^cells.

**Table 1:** EPO biological activity *in vitro*

Culture	EPO ELISA (mIU/ml)	EPO activity assay (TF-1) (mIU/ml)	Activity ratio (activity/protein)
Mock BALB/3T3	0	0	-
Transduced BALB/3T3	10.74 ± 0.78	373.3 ± 37.12*	×35
Mock neonatal fibroblasts	0	0	-
Transduced neonatal fibroblasts	51.24 ± 11.17	576.7 ± 186.7*	×12

RhEPO protein levels: the presence of rhEPO in transduced culture supernants was analyzed by a commercial ELISA kit. RhEPO biologic activity (TF-1 cell proliferation assay): EPO biological activity was analyzed using the EPO-sensitive human erythroleukemia cell line TF-1, in which proliferation and metabolic activity is EPO-dependent. *Significantly more active than rhEPO standard (Eprex), *p *< 0.05.

To generate viable cardiac cell culture producing rhEPO for subsequent transplantation, we used rat neonatal cardiac fibroblasts. EPO activity parameters of transduced cardiac fibroblasts are presented in Table 1. The produced protein was found to be 12 times more active than recombinant protein (Eprex; *p *= 0.03). The rhEPO production level in transduced cells was 14.2 ± 3.1 mIU/24 h/10^6 ^cells.

### Anti-apoptotic effect of EPO in isolated cardiomyocytes

**Figure 1:**
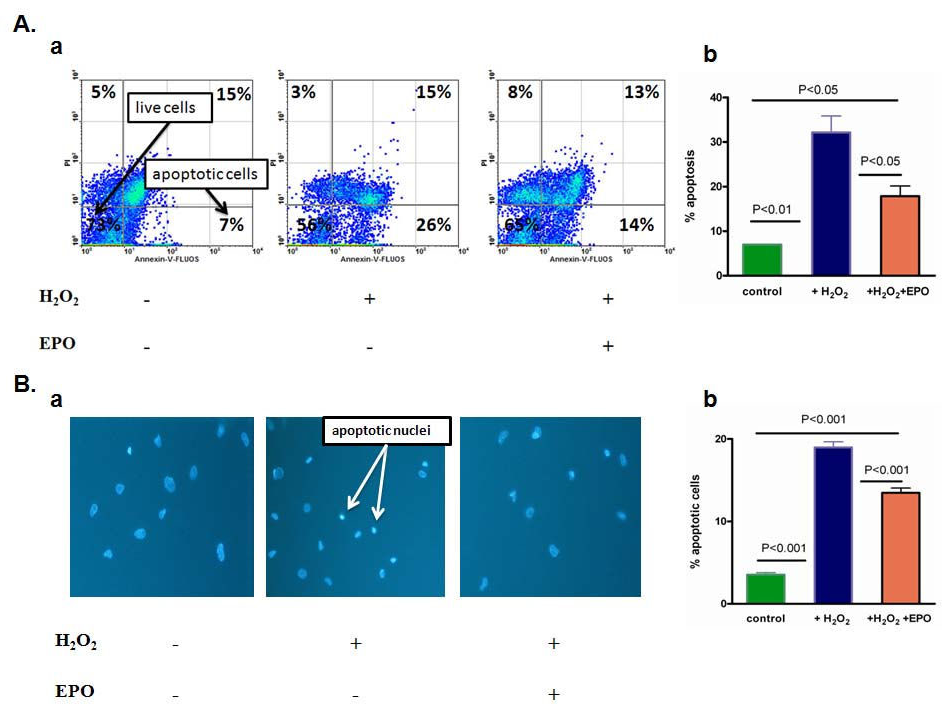
**RhEPO inhibits H_2_O_2_-induced apoptosis in isolated cardiomyocytes.** Neonatal rat cardiomyocytes were treated with PBS as a control or with H_2_O_2 _(150 μM) for 60 minutes in the 24 h presence or absence of rhEPO produced from transduced fibroblasts (12 mIU/ml). **(A) **Annexin V/PI staining: (a) Representative density profile (percentage of cells in each quadrant is indicated); (b) Quantitative analysis, showing that EPO attenuated cardiomyocyte apoptosis. **(B) **DAPI staining: (a) representative photomicrographs (arrows show apoptotic cardiomyocyte nuclei (chromatin condensation and nuclei fragmentation); (b) quantitative analysis, as represented by relative apoptotic nuclei counts.

The rate of apoptosis was significantly lower in cultured cardiomyocytes treated with supernants derived from rhEPO-tranduced fibroblasts. To determine the protective effect of rhEPO produced from genetically modified cardiac fibroblasts on cardiomyocyte apoptosis, we used an H_2_O_2_-induced oxidative stress model [24]. Isolated rat neonatal cardiomyocytes were treated with 150 μM H_2_O_2 _for 60 minutes, with or without 24 h preconditioning with supernants containing rhEPO (12 mIU/ml) from transduced cardiac fibroblasts. The degree of apoptosis was assayed by Annexin V/PI staining and morphologically evaluated using a DAPI nuclear stain. FACS analysis of Annexin V/PI staining showed that the percentage of apoptotic cells was significantly lower in cardiomyocytes pretreated with rhEPO-containing supernants compared with control (Figure 1A): 17.8 ± 2.3% versus 32.1 ± 3.7% apoptotic cells (*p *< 0.05).

Morphological changes in cells that were exposed to oxidative stress were evaluated after DAPI staining. Apoptotic nuclei counts showed a significant reduction in cell death in cells treated with rhEPO-containing supernants, compared to untreated cells: 13.5 ± 0.6% versus 19.0 ± 0.7%, *p *< 0.001 (Figure 1B).

### *In situ *expression of erythropoietin promotes cytoprotection and vascularization

**Figure 2:**
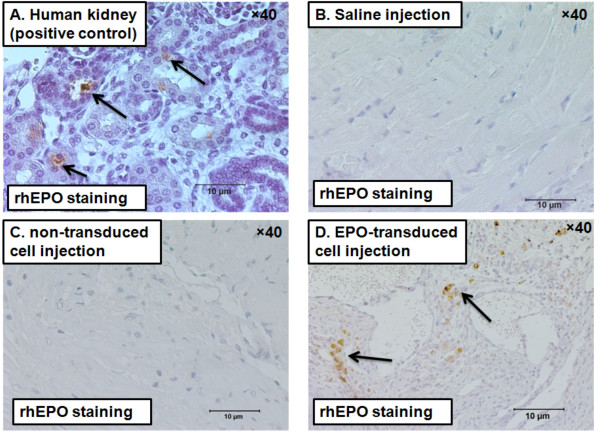
**Photomicrographs of heart sections, immunostained with anti-human erythropoietin antibodies (brown), 4 weeks after cell transplantation.(A)** Human kidney positive control.** (B)** saline-injected heart. **(C)** Non-transduced fibroblast-transplanted heart. **(D)** RhEPO in transduced fibroblast-transplanted heart (infarct zone). Arrows show rhEPO expression.

There was no difference among the groups in the levels of hematocrit at all time points (Table 2). Four weeks after cell transplantation, heart tissue sections were stained with anti-rhEPO monoclonal antibodies to detect rhEPO expression from implanted cells. Human kidney tissue was used as positive control (Figure 2A). RhEPO was detected only in hearts treated with transduced cardiac fibroblasts 5 weeks post MI (Figure 2B–D). The staining was identified in all animals treated with transduced cells (n = 5), towards the center of the infarct zone.

**Table 2:** Serial hematocrit measurements in rats after MI, showing that EPO-transduced cell injection does not increase systemic hematocrit levels

Treatment time	Saline (n = 9)	EPO-transduced cells (n = 9)	Non-transduced cells (n = 9)
Baseline (before injection; %)	47.5 ± 0.3	47.2 ± 0.2	47.2 ± 0.1
1 week post-injection (%)	47.4 ± 0.2	47.0 ± 0.1	47.39 ± 0.2
4 weeks post-injection (%)	47.4 ± 0.18	46.8 ± 0.2	47.15 ± 0.2

*p *= 1.00.

**Figure 3:**
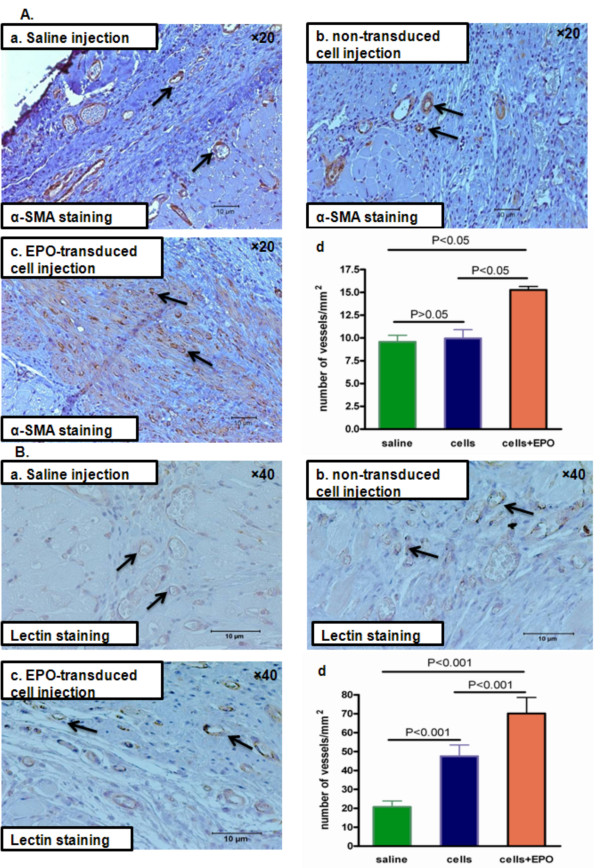
**Four weeks after cell transplantation, rhEPO expression promotes neo-angiogenesis *****in vivo***. **(A) **α-Smooth muscle actin staining (brown) in infarcted hearts (infarct border zones). Arrows show stained blood vessels. (a) Saline injection; (b) non-transduced cell injection; (c) rhEPO-transduced cell injection; (d) quantitative analysis. **(B)** Lectin (BS-1) staining (brown) in infarcted hearts (infarct border zones). Arrows show stained blood vessels. (a) Saline injection; (b) non-transduced cell injection; (c) rhEPO-transduced cell injection; (d) quantitative analysis.

The degree of vascularization was evaluated by counting capillary blood vessels in the infarct border zones using α-smooth muscle actin and lectin stainings for vascular endothelium localization. Compared with non-transduced fibroblasts and saline, implanted rhEPO-producing fibroblasts enhanced angiogenesis, as shown in both α-smooth muscle actin (Figure 3A; 15.27 ± 0.37 versus 9.93 ± 0.97 versus 9.57 ± 0.72 vessels/mm^2^, respectively, *p *< 0.05), and lectin stained sections (Figure 3B; 70.1 ± 8.5 versus 47.5 ± 5.9 versus 20.7 ± 3.6 vessels/mm^2^, respectively, *p *< 0.05).

**Figure 4:**
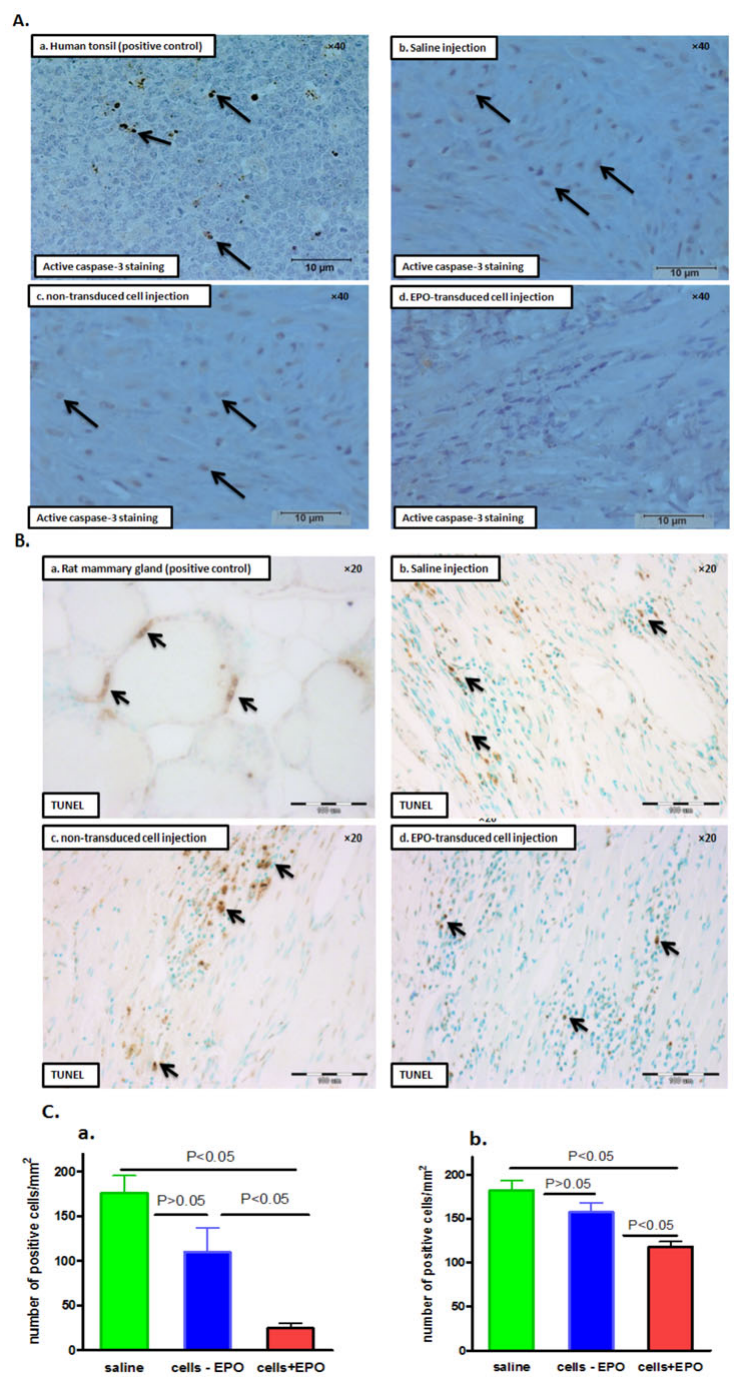
**Four weeks after cell transplantation, rhEPO expression attenuates cardiac cell apoptosis *****in vivo***. **(A) **Representative photomicrographs of active caspase-3 staining (brown) in infarcted hearts (infarct border zone). Arrows show nuclear active caspase-3 staining. (a) Human tonsil tissue (positive control); (b) saline injection; (c) non-transduced cell injection; (d) rhEPO-transduced cell injection. **(B)** Representative photomicrographs of TUNEL staining (brown) in infarcted hearts (infarct border zone). (a) Rat mammary gland tissue (positive control); (b) saline injection; (c) non-transduced cell injection; (d) rhEPO-transduced cell injection. **(C)** Quantitative analysis of apoptosis in infarcted hearts. (a) active caspase-3 staining; (b) TUNEL.

Apoptosis in infarcted hearts was detected by active caspase-3 staining with polyclonal antibodies detecting only the active (effector) form of caspase-3 (Figure 4A) [25,26]. Human tonsil tissue was used as a positive control (Figure 4A, a). RhEPO-transduced cell transplantation resulted in a significant decrease in the number of active-caspase-3 positive cells (24 ± 6 versus 176 ± 20 versus 110 ± 27 cells per mm^2 ^in saline and non-transduced cell injections, *p *< 0.05; Figure 4C, a). The staining revealed mainly nuclear localization of active caspase-3 (Figure 4A) [26]. Apoptosis was also evaluated by commercially available TUNEL kit (Figure 4B). Rat mammary gland tissue served as a positive control (Figure 4B, a). Similar to active caspase-3 staining, TUNEL staining showed that rhEPO-transduced cell transplantation resulted in a significant decrease in the number of apoptotic cells (118 ± 6 versus 182 ± 12 versus 157 ± 10 cells per mm^2 ^in saline and non-transduced cell injections, *p *< 0.05; Figure 4C, b). These results suggest that local rhEPO expression prevented cardiac cell apoptosis *in vivo*.

### EPO-transduced cell injection did not improve LV remodeling and function

To examine potential cardioprotective effects of rhEPO-producing fibroblasts *in vivo*, we used a rat model of AMI, which resulted in progressive LV remodeling and dysfunction [19]. Overall, 45 rats were included in the final transplantation study. Rats were randomized into three treatment groups (n = 15 in each group): transduced rhEPO-producing cells, non-transduced cells, and saline injection. Ten animals from each group (five additional animals in each group were sacrificed one month after transplantation for immunohistochemical examination) were assessed with echocardiography.

Serial echocardiography tests showed that EPO-transduced and non-transduced cell injection did not reduce LV remodeling and dysfunction 5 and 9 weeks post MI (Table 3). At 5 weeks, there was a trend towards more scar thinning in the EPO-treated group (anterior wall diastolic thickness, *p *= 0.07; LV end diastolic dimension, *p *= 0.08).

**Table 3:** Comparison of LV remodeling and function between EPO-transduced, non-transduced cell or saline injections by two-dimensional echocardiography, before (baseline), or 5 or 9 weeks after MI

	EPO-transduced cells (n = 10)	Non-transduced cells (n = 9)	Saline (n = 9)	*p*^†^
**5-week follow-up**				
Heart rate				0.17
Baseline	308.8 ± 12.6	273.8 ± 11.7	273.0 ± 11.6	
5 weeks	252.7 ± 9.5	252.5 ± 14.0	262.8 ± 13.5	
p*	0.0068	0.32	0.55	
				
AW d (cm)				0.07
Baseline	0.13 ± 0.01	0.17 ± 0.007	0.13 ± 0.01	
5 weeks	0.09 ± 0.006	0.10 ± 0.01	0.10 ± 0.01	
p*	0.006	0.0002	0.04	
				
LVEDD				0.08
Baseline	0.76 ± 0.02	0.72 ± 0.02	0.82 ± 0.02	
5 weeks	1.06 ± 0.02	0.98 ± 0.03	1.00 ± 0.03	
p*	<0.0001	<0.0001	0.0009	
				
LVESD				0.22
Baseline	0.60 ± 0.03	0.56 ± 0.02	0.64 ± 0.03	
5 weeks	0.88 ± 0.03	0.81 ± 0.04	0.80 ± 0.04	
p*	<0.0001	<0.0001	<0.0001	
				
LVEDA				0.34
Baseline	0.39 ± 0.03	0.35 ± 0.01	0.43 ± 0.02	
5 weeks	0.73 ± 0.04	0.65 ± 0.04	0.67 ± 0.05	
p*	<0.0001	<0.0001	0.0011	
				
LVESA				0.36
Baseline	0.24 ± 0.02	0.21 ± 0.01	0.26 ± 0.02	
5 weeks	0.52 ± 0.04	0.42 ± 0.04	0.44 ± 0.05	
p*	<0.0001	<0.0001	0.0004	
				
FS (%)				0.99
Baseline	20.6 ± 2.9	22.3 ± 1.7	25.07 ± 2.2	
5 weeks	16.2 ± 1.4	17.8 ± 1.6	20.96 ± 2.0	
p*	0.14	0.008	0.002	
				
**9-week follow-up**				
Heart rate				0.28
Baseline	303.2 ± 12.7	274.4 ± 13.3	273.0 ± 11.6	
9 weeks	242.0 ± 6.3	235.5 ± 12.2	246.2 ± 7.7	
p*	0.04	0.12	0.0014	
				
AW d (cm)				0.19
Baseline	0.13 ± 0.01	0.17 ± 0.007	0.13 ± 0.01	
9 weeks	0.09 ± 0.01	0.12 ± 0.01	0.11 ± 0.01	
p*	0.03	0.0002	0.3	
				
LVEDD				0.22
Baseline	0.76 ± 0.02	0.72 ± 0.02	0.82 ± 0.02	
9 weeks	1.11 ± 0.04	1.03 ± 0.03	1.05 ± 0.03	
p*	<0.0001	<0.0001	0.0003	
				
LVESD				0.3
Baseline	0.60 ± 0.03	0.56 ± 0.02	0.64 ± 0.03	
9 weeks	0.95 ± 0.05	0.84 ± 0.04	0.86 ± 0.05	
p*	<0.0001	<0.0001	<0.0001	
				
LVEDA				0.42
Baseline	0.39 ± 0.03	0.35 ± 0.01	0.43 ± 0.02	
9 weeks	0.83 ± 0.06	0.71 ± 0.05	0.74 ± 0.06	
p*	<0.0001	<0.0001	0.0006	
				
LVESA				0.37
Baseline	0.24 ± 0.02	0.21 ± 0.01	0.26 ± 0.02	
9 weeks	0.61 ± 0.06	0.48 ± 0.05	0.51 ± 0.06	
p*	0.0001	0.0001	0.0003	
				
FS (%)				0.09
Baseline	20.6 ± 2.9	22.3 ± 1.72	25.1 ± 2.2	
9 weeks	14.3 ± 1.8	18.7 ± 1.73	19.8 ± 2.4	
p*	0.05	0.02	0.0008	

AW d, anterior wall diastolic thickness; LVEDD, LV end diastolic dimension; LVESD, LV end systolic dimension; LVEDA, LV end diastolic area; LVESA, LV end systolic area; FS, LV fractional shortening [(LVIDd – LVIDs)/LVIDd] × 100. Values are mean ± SEM; p*, *p*-values derived from paired comparisons between baseline and 5/9 week measurements; p^†^, *p*-values for the differences between groups over time (two-way repeated-measures ANOVA, *p*-value for interaction).

Morhpometric analysis of the infarcted hearts showed that EPO-transduced cell injection also fails to prevent infarct expansion and scar thinning (Table 4). For major parameters (average scar thickness, LV cavity area, whole LV area and expansion index), EPO-transduced cells showed significant adverse remodeling compared to non-transduced cells with a magnitude similar to saline-treated animals. Furthermore, non-transduced cell injection attenuated infarct expansion and preserved LV area, compared to saline-treated animals.

**Table 4:** Comparison of LV remodeling by postmortem morphometry, 9 weeks after MI

	EPO-transduced cells (n = 10)	Non-transduced cells (n = 9)	Saline (n = 9)	p* ANOVA
LV maximal diameter (mm)	8.2 ± 0.4	6.2 ± 0. 31	8.1 ± 0.33	<0.0001
*p *(Bonferroni test)	<0.001^†^	<0.001^‡^	-	
				
Average wall thickness (mm)	1.4 ± 0.05	1.2 ± 0.04	1.4 ± 0.03	0.003
*p *(Bonferroni test)	<0.05^†^	<0.05^‡^	-	
				
Average scar thickness (mm)	0.62 ± 0.03	0.92 ± 0.04	1.0 ± 0.05	<0.0001
*p *(Bonferroni test)	<0.001^§^	-	-	
				
Relative scar thickness	0.46 ± 0.05	0.59 ± 0.03	0.56 ± 0.05	0.22
*p *(Bonferroni test)	-	-	-	
				
Muscle area (mm^2^)	43 ± 1.4	43 ± 4.2	47 ± 2.3	0.55
*p *(Bonferroni test)	-	-	-	
				
LV cavity area (mm^2^)	57 ± 8.0	40 ± 4.9	65 ± 5.3	0.002
*p *(Bonferroni test)	<0.05^†^	<0.01^‡^	-	
				
Whole LV area (mm^2^)	110 ± 7.5	77 ± 7.0	110 ± 7.0	<0.0001
*p *(Bonferroni test)	< 0.001^†^	< 0.001^‡^	-	
				
Expansion index	1.1 ± 0.08	0.89 ± 0.05	1.2 ± 0.13	0.04
*p *(Bonferroni test)	-	<0.05^‡^	-	
				
Epicardial scar length (mm)	7.2 ± 1.2	6.5 ± 1.2	8.1 ± 1.6	0.68
*p *(Bonferroni test)	-	-	-	
				
Endocardial scar length (mm)	7.3 ± 1.6	6.2 ± 1.2	6.6 ± 1.0	0.65
*p *(Bonferroni test)	-	-	-	

Values are means ± SEM. *p *(Bonferroni test), *p*-values from Bonferroni multiple comparison test; p*, *p*-values for the differences between groups (one-way ANOVA). ^†^Versus non-transduced cells; ^‡^versus saline; ^§^versus saline and non-transduced cells.

## Discussion

To the best of our knowledge, the present study is the first to use reprogrammed cardiac fibroblasts encoding the rhEPO gene to repair infarcted myocardium *in situ*. In previous experiments, rhEPO was delivered systemically prior to, at different time points and immediately after ischemia/reperfusion or cardiomyopathy induction [27-30]. In our study, rhEPO produced from genetically modified cardiac fibroblasts exhibited a strong anti-apoptotic effect *in vitro*, promoted angiogenesis and attenuated apoptosis in the infarcted tissue. However, despite positive *in vitro *results and cytoprotection of EPO-transduced cells in the infarcted hearts, restoration of LV function was not observed, and there was a trend to more adverse remodeling.

Cardiac fibroblasts present an attractive gene carrier into the infarcted heart. Although not an 'ideal' source for transplantation (due to lack of contractility and so on), fibroblasts, constituting the major cell population of the myocardium, can be easily obtained from clinically accessible sites, expanded *in vitro *without significant limitation, and, unlike cardiomyocytes, can be transduced highly efficiently by retroviral EPO-based gene transfer.

Our findings concur with other studies that show a lack of functional improvement following EPO treatment despite positive cellular effects (apoptosis inhibition and angiogenesis) [31-35], as well as one clinical trial conducted with EPO analogue darbepoetin alfa [36].

The reason for the inconsistency between the cytoprotective effect of EPO *in vitro *and *in situ *versus the lack of functional improvement *in vivo *is unclear. A possible explanation is that an EPO therapeutic effect on LV remodeling and function is mainly mediated by endothelial progenitor cell recruitment, which is achieved by systemic administration [34,37,38]. This notion is supported by the positive correlation found between EPO plasma levels and endothelial progenitor cell recruitment in an observational study of patients after acute MI [39]. Thus, local EPO therapy is insufficient for functional improvement.

In addition, the lack of improvement could be related to the timing of therapy. In most of the positive EPO studies systemic cytokine administration was initiated before or immediately after ischemia/reperfusion induction. We used a more severely injured model with more clinically relevant timing of cell delivery (1 week post MI). However, at this stage, EPO could be less effective due to a relatively low cell apoptotic rate and the lack of stem cell homing signals from the infarct [35].

### Study limitations

Cell engraftment and survival was not quantified. Bromodeoxyuridine (BrdU) or other cellular markers were not used to avoid interference with cell capacity to colonize and proliferate. Such interference could cause a decrease in the proposed EPO effect.

Retroviral transduction itself can potentially alter the phenotype of the transduced cells. However, the expression profile of transduced cells did not exhibit any negative effects when the transduced cell culture supernants were added to cardiomyocyte culture *in vitro*.

In order to avoid possible systemic interference, we did not apply an immunosuppression protocol. However, EPO detection 1 month after cell transplantation suggests that immune response, if evoked, is limited in magnitude. The EPO tissue level could not be quantified due to technical constraints for detection of predicted low levels of the protein.

## Conclusion

*In situ *expression of rhEPO by genetically engineered cardiac fibroblasts exerts cytoprotective and pro-angiogenic effects. However, these favorable effects were not translated into functional improvement after MI. The results suggest that for successful local EPO-based therapy, several improvements should be made to determine the optimal cell type for gene delivery, the most effective recombinant viral vector, myocardial EPO response and, notably, an accurate therapeutic window for cell and gene transfer.

## Competing interests

The authors declare that they have no competing interests.

## Authors' contributions

ER carried out the *in vitro *and *in vivo *experiments, including data acquisition and analysis. OS participated in study design and coordination, and helped in drafting the manuscript. AN participated in conceptual study design and coordination, and helped to draft the manuscript. TE provided help in study design, data acquisition and analysis. MF participated in data acquisition and analysis. RH carried out the *in vivo *experiments. AD participated in conceptual study design and coordination, and helped to draft the manuscript. JL conceived the study, participated in its design and coordination, and helped to draft the manuscript.
